# *In vitro* and *in vivo* activity of cefiderocol against *Achromobacter* spp. and *Burkholderia cepacia* complex, including carbapenem-non-susceptible isolates

**DOI:** 10.1128/aac.00346-23

**Published:** 2023-11-16

**Authors:** Miki Takemura, Rio Nakamura, Merime Ota, Ryuichiro Nakai, Daniel F. Sahm, Meredith A. Hackel, Yoshinori Yamano

**Affiliations:** 1 Laboratory for Drug Discovery and Disease Research, Shionogi & Co., Ltd., Osaka, Japan; 2 Department of Biofunctional Evaluation ΙI, Shionogi TechnoAdvance Research & Co., Ltd., Osaka, Japan; 3 International Health Management Associates, Schaumburg, Illinois, USA; Columbia University Irving Medical Center, New York, USA

**Keywords:** *Achromobacter *spp., *Burkholderia cepacia *complex, carbapenem non-susceptible, cefiderocol, multidrug resistant

## Abstract

*Achromobacter* spp. and *Burkholderia cepacia* complex (Bcc) are rare but diverse opportunistic pathogens associated with serious infections, which are often multidrug resistant. This study compared the *in vitro* antibacterial activity of the siderophore antibiotic cefiderocol against *Achromobacter* spp. and Bcc isolates with that of other approved antibacterial drugs, including ceftazidime-avibactam, ciprofloxacin, colistin, imipenem-relebactam, and meropenem-vaborbactam. Isolates were collected in the SIDERO multinational surveillance program. Among 334 *Achromobacter* spp. isolates [76.6% from respiratory tract infections (RTIs)], cefiderocol had minimum inhibitory concentration (MIC)_50/90_ of 0.06/0.5 µg/mL overall and 0.5/4 µg/mL against 52 (15.6%) carbapenem-non-susceptible (Carb-NS) isolates. Eleven (3.3%) *Achromobacter* spp. isolates overall and 6 (11.5%) Carb-NS isolates were not susceptible to cefiderocol. Among 425 Bcc isolates (73.4% from RTIs), cefiderocol had MIC_50/90_ of ≤0.03/0.5 µg/mL overall and ≤0.03/1 µg/mL against 184 (43.3%) Carb-NS isolates. Twenty-two (5.2%) Bcc isolates overall and 13 (7.1%) Carb-NS isolates were not susceptible to cefiderocol. Cumulative MIC distributions showed cefiderocol to be the most active of the agents tested *in vitro* against both *Achromobacter* spp. and Bcc. In a neutropenic murine lung infection model and a humanized pharmacokinetic immunocompetent rat lung infection model, cefiderocol showed significant bactericidal activity against two meropenem-resistant *Achromobacter xylosoxidans* strains compared with untreated controls (*P* < 0.05) and vehicle-treated controls (*P* < 0.05), respectively. Meropenem, piperacillin-tazobactam, ceftazidime, and ciprofloxacin comparators showed no significant activity in these models. The results suggest that cefiderocol could be a possible treatment option for RTIs caused by *Achromobacter* spp. and Bcc.

## INTRODUCTION

*Achromobacter* spp. and *Burkholderia* spp. are diverse and important opportunistic pathogens (1) that are increasingly found in patients with cystic fibrosis (CF) (2, 3) and are frequent causes of infection following lung transplantation (3–5). Common *Achromobacter* infections in individuals without CF are pneumonia and bacteremia, which are generally hospital acquired (3, 6), although many bloodstream infections have a community onset (7). Infections caused by *Burkholderia pseudomallei*, which is endemic in tropical and subtropical regions, are often associated with considerable morbidity and mortality (8).

*Achromobacter* spp. and *Burkholderia* spp. infections are therapeutically challenging due to their often multidrug-resistant (MDR) nature (2). Resistance mechanisms for *Achromobacter* spp. (9) occur mainly via mutations in (intrinsic) multidrug efflux pumps and (chromosomal) Ambler Class C and acquired Class D β-lactamases (1, 3). The lesser role of serine carbapenemase production in resistance reduces the potential benefit of newer β-lactam/β-lactamase inhibitor combinations (e.g., ceftazidime/avibactam, imipenem/relebactam) (3). *Burkholderia* spp. exhibit intrinsic and acquired drug resistance, mainly via outer membrane permeability, including restrictive porin channels, combined with efflux pumps (10), and periplasmic or membrane-bound β-lactamases (1, 11). Clinical isolates of *B. cepacia* have been shown to harbor the *bla*_TEM_, *bla*_CTX-M_, *bla*_CTX-M-15_, *bla*_OXA_, *bla*_SHV-12_, and *bla*_VEB_ genes (12).

There are currently no standard treatment recommendations for *Achromobacter* spp. or *Burkholderia* spp. infections, and treatment is based on *in vitro* susceptibilities (1). Piperacillin-tazobactam, carbapenems, ceftazidime, and trimethoprim-sulfamethoxazole are suggested as first-line treatments for *Achromobacter* spp. infections in patients with CF (1). Trimethoprim-sulfamethoxazole, meropenem, and ceftazidime are suggested first-line treatments for *Burkholderia cepacia* complex (Bcc) infections (1). Among non-CF patients with Bcc bacteremia, no single antibiotic regimen, which included meropenem, ceftazidime, tigecycline, levofloxacin, or trimethoprim-sulfamethoxazole, was associated with improved survival, and combination therapy did not reduce mortality (13).

Cefiderocol has potent *in vitro* activity against Gram-negative pathogens, including Enterobacterales and non-fermenting bacteria such as *Pseudomonas aeruginosa, Stenotrophomonas maltophilia*, and *Acinetobacter baumannii* (14). It has also shown *in vitro* activity against clinical isolates of *Achromobacter* spp. from patients with CF (15) and of *B. pseudomallei* from patients with mainly blood, lung, skin, and soft tissue infections (8).

In this study, we compared the *in vitro* and *in vivo* antibacterial activity of cefiderocol with that of other approved antibacterial drugs against *Achromobacter* spp. and Bcc isolates, including a large collection of isolates from the 5-year, multinational SIDERO surveillance studies from patients hospitalized with serious infections (16, 17).

## RESULTS

### Isolates

Among 334 isolates of *Achromobacter* spp. collected between 2015 and 2019 globally, the most common was *Achromobacter xylosoxidans* (*n* = 311, 93.1%) followed by *Achromobacter insolitus* (*n* = 11, 3.3%), *Achromobacter* spp. (*n* = 9, 2.7%), *Achromobacter denitrificans* (*n* = 2, 0.6%), and *Achromobacter piechaudii* (*n* = 1, 0.3%). Most (53.0%) isolates were from North America, followed by Latin America (13.8%) and Asia (13.5%) (Table S1). Fifty-two (15.6%) isolates were carbapenem non-susceptible (Carb-NS), which were found most frequently in North America (65.4%), Latin America (11.5%), and the South Pacific (11.5%). Most isolates were from respiratory tract infections (RTIs), both overall (76.6%) and for Carb-NS isolates (92.3%) (Table 1). Carb-NS isolates were also found in skin infections (3.8%) and bloodstream infections (1.9%) (Table 1).

**TABLE 1:** Body site of overall, carbapenem-susceptible, and carbapenem-non-susceptible *Achromobacter* spp. and *Burkholderia cepacia* complex isolates^*f*^

Source	*Achromobacter* spp. infections			*Burkholderia cepacia* complex infections		
	Total (%)	Carbapenem-susceptible (%)*^a^*	Carbapenem non-susceptible (%)*^b^*	Total (%)	Carbapenem-susceptible (%)*^a^*	Carbapenem non-susceptible (%)*^b^*
**All**	**334** (**100**)	**282 (84.4^*c*^**)	**52 (15.6^*c*^**)	**425** (**100**)	**241 (56.7^*c*^**)	**184 (43.3^*c*^**)
Respiratory tract	256 (76.6)	208 (73.8)	48 (92.3)	312 (73.4)	168 (69.7)	144 (78.3)
Blood	24 (7.2)	23 (8.2)	1 (1.9)	56 (13.2)	34 (14.1)	22 (12.0)
Skin and skin structure/wound	20 (6.0)	18 (6.4)	2 (3.8)	29 (6.8)	19 (7.9)	10 (5.4)
GI tract	16 (4.8)	16 (5.7)	0 (0)	5 (1.2)	4 (1.7)	1 (0.5)
Urinary tract	13 (3.9)	13 (4.6)	0 (0)	22 (5.2)	16 (6.6)	6 (3.3)
Unknown/other	5^*d*^ (1.5)	4 (1.4)	1 (1.9)	1^*e*^ (0.2)	0	1^*e*^ (0.5)

^*a*^ As a proportion of all carbapenem-susceptible isolates, unless otherwise specified.

^*b*^ As a proportion of all carbapenem-non-susceptible isolates, unless otherwise specified.

^*c*^ As a proportion of the total number of isolates of the species specified.

^*d*^ Unknown.

^*e*^ Thoracentesis.

^*f*^ GI, gastrointestinal.

Among 425 Bcc isolates collected, the most common species was *B. cepacia* (*n* = 202, 47.5%), followed by *Burkholderia cenocepacia* (*n* = 107, 25.2%) and *Burkholderia multivorans* (*n* = 98, 23.1%). Most (53.9%) isolates were from Europe, including Spain (12.0%) and Czech Republic (9.2%) (Table S1). Nearly half (*n* = 184, 43.3%) of the isolates were Carb-NS, which were found most frequently in North America (46.7%), Spain (16.8%), and the Czech Republic (8.2%). Most isolates were from RTIs, both overall (73.4%) and Carb-NS isolates (78.3%) (Table 1). Carb-NS isolates were also found in bloodstream infections (12.0%), skin infections (5.4%), and urinary tract infections (3.3%) (Table 1).

### *In vitro* activity

**Fig 1:**
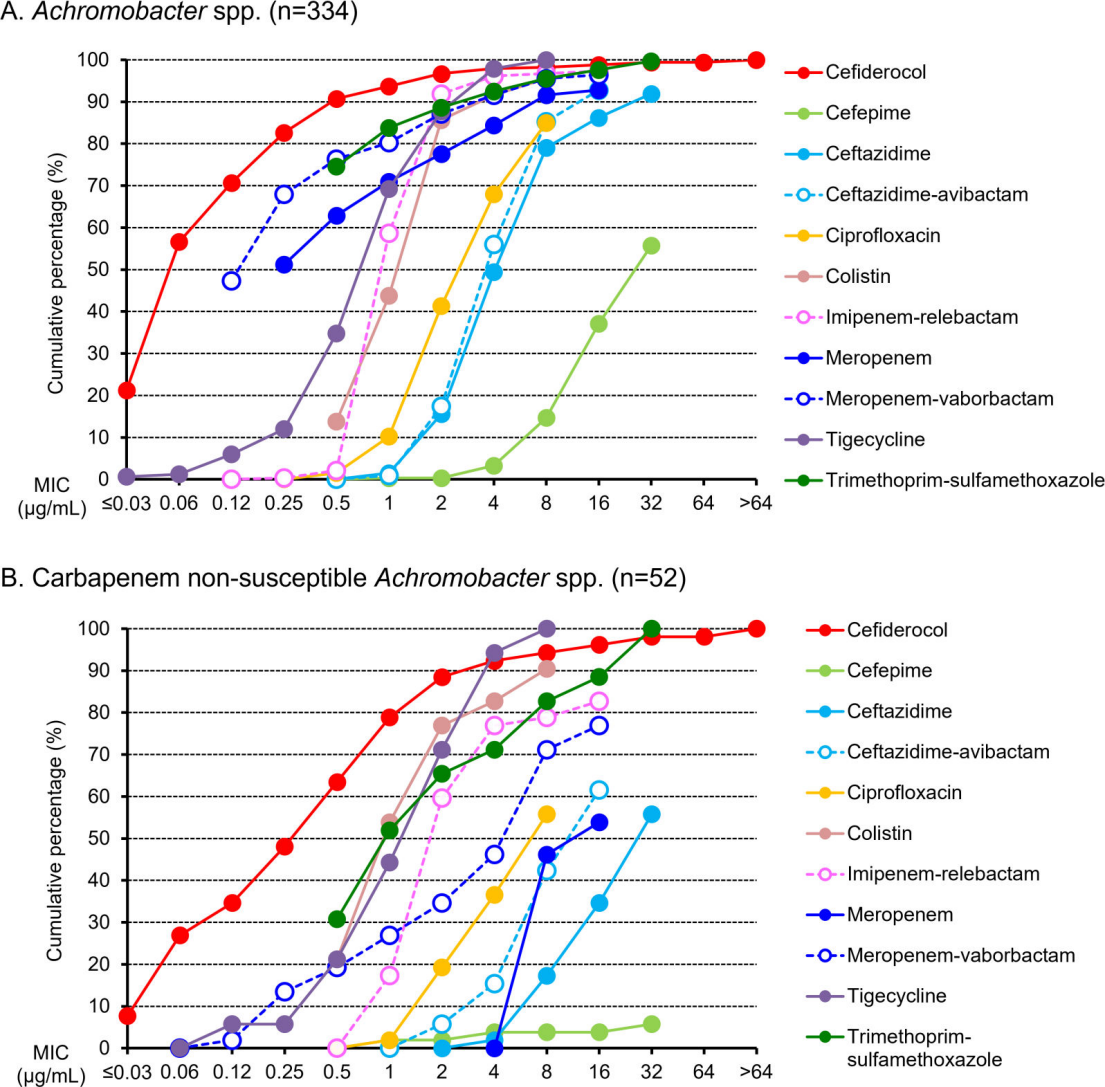
Cumulative percentage MIC distributions of cefiderocol and comparators against: (A) *Achromobacter* spp.; (B) carbapenem-non-susceptible *Achromobacter* spp.

Against 334 isolates of *Achromobacter* spp., cefiderocol showed *in vitro* activity with MIC_50/90_ of 0.06/0.5 µg/mL (Table 2). Only 11 (3.3%) isolates had MIC >2 µg/mL [MIC = 4 µg/mL: *n* = 4 (1.2%); MICs >4 µg/mL: *n* = 7 (2.1%)], considered not susceptible based on European Committee on Antimicrobial Susceptibility Testing (EUCAST) pharmacokinetic (PK)/pharmacodynamic (PD) breakpoints (Table 2). According to cumulative MIC distributions, cefiderocol was the most active of the agents tested against *Achromobacter* spp. (Fig. 1A). Against Carb-NS isolates, the MIC_50_ and MIC_90_ of cefiderocol were 0.5 and 4 µg/mL, respectively (Table 2). Cefiderocol inhibited the growth of 92.3% (48/52) of Carb-NS *Achromobacter* spp. at a concentration of 4 µg/mL (Fig. 1B). Six (11.5%) isolates were not susceptible to cefiderocol based on EUCAST PK/PD breakpoints (Table 2).

**TABLE 2:** *In vitro* activity of cefiderocol and comparators against *Achromobacter* spp. and carbapenem-non-susceptible *Achromobacter* spp. collected globally from 2015 to 2019^*l*^

Phenotype (no. of isolates)	Antimicrobial agent	MIC (µg/mL)				Susceptibility rate (%)	
		*N*	Range	MIC_50_	MIC_90_	CLSI	EUCAST
All isolates	Cefiderocol	334	≤0.03 to >64	0.06	0.5	NA	96.7^*a*^
	Cefepime	334	1 to >32	32	>32	14.7^*b*^	3.3^*c*^
	Ceftazidime	334	1 to >32	8	32	79.0^*b*^	49.4^*c*^
	Ceftazidime-avibactam	334	1 to >16	4	16	NA	85.3^*d*^
	Ciprofloxacin	334	0.5 to >8	4	>8	10.2^*e*^	0^*f*^
	Colistin	334	≤0.25 to >8	2	4	NA	NA
	Imipenem-relebactam	334	0.25 to 16	1	2	NA	91.9^*a*^
	Meropenem	334	≤0.12 to >16	0.25	8	84.4^*g*^	71.0^*h*^
	Meropenem-vaborbactam	334	≤0.06 to >16	0.25	4	NA	95.5*^d^*
	Tigecycline	334	≤0.03 to 8	1	4	NA	34.7^*i*^
	Trimethoprim-sulfamethoxazole	334	≤0.25 to >32	0.5	4	88.6^*j*^	NC
Meropenem non-susceptible^*k*^	Cefiderocol	52	≤0.03 to >64	0.5	4	NA	88.5^*a*^
	Cefepime	52	1 to >32	>32	>32	3.8^*b*^	3.8^*c*^
	Ceftazidime	52	4 to >32	32	>32	17.3^*b*^	1.9^*c*^
	Ceftazidime-avibactam	52	2 to >16	16	>16	NA	42.3^*d*^
	Ciprofloxacin	52	1 to >8	8	>8	1.9^*e*^	0^*f*^
	Colistin	52	≤0.25 to >8	1	8	NA	NA
	Imipenem-relebactam	52	1 to >16	2	>16	NA	59.6^*a*^
	Meropenem	52	8 to >16	16	>16	0^*g*^	0^*h*^
	Meropenem-vaborbactam	52	0.12 to >16	8	>16	NA	71.2^*d*^
	Tigecycline	52	0.12 to 8	2	4	NA	21.2^*i*^
	Trimethoprim-sulfamethoxazole	52	≤0.25 to 32	1	32	65.4^*j*^	NC

^*a*^ EUCAST non-species PK/PD breakpoint: susceptible: ≤2 μg/mL.

^*b*^ CLSI breakpoint for other non-Enterobacterales: susceptible: ≤8 μg/mL.

^*c*^ EUCAST non-species PK/PD breakpoint: susceptible: ≤4 μg/mL.

^*d*^ EUCAST non-species PK/PD breakpoint: susceptible: ≤8 μg/mL.

^*e*^ CLSI breakpoint for other non-Enterobacterales: susceptible: ≤1 μg/mL.

^*f*^ EUCAST non-species PK/PD breakpoint: susceptible: ≤0.25 μg/mL.

^*g*^ CLSI breakpoint for other non-Enterobacterales: susceptible: ≤4 μg/mL.

^*h*^ EUCAST *A. xylosoxidans* breakpoint: susceptible: ≤1 μg/mL.

^*i*^ EUCAST non-species PK/PD breakpoint: susceptible: ≤0.5 μg/mL.

^*j*^ CLSI breakpoint for other non-Enterobacterales: susceptible: ≤2 μg/mL.

^*k*^ Meropenem non-susceptible: meropenem MIC ≥8 μg/mL by CLSI.

^*l*^ CLSI, Clinical and Laboratory Standards Institute [18]; EUCAST, European Committee on Antimicrobial Susceptibility Testing [19]; NA, no breakpoint available; NC, susceptibility rate could not be calculated because the lower limit of the MIC measurement was 0.25 μg/mL, which is higher than the susceptibility breakpoint.

**Fig 2:**
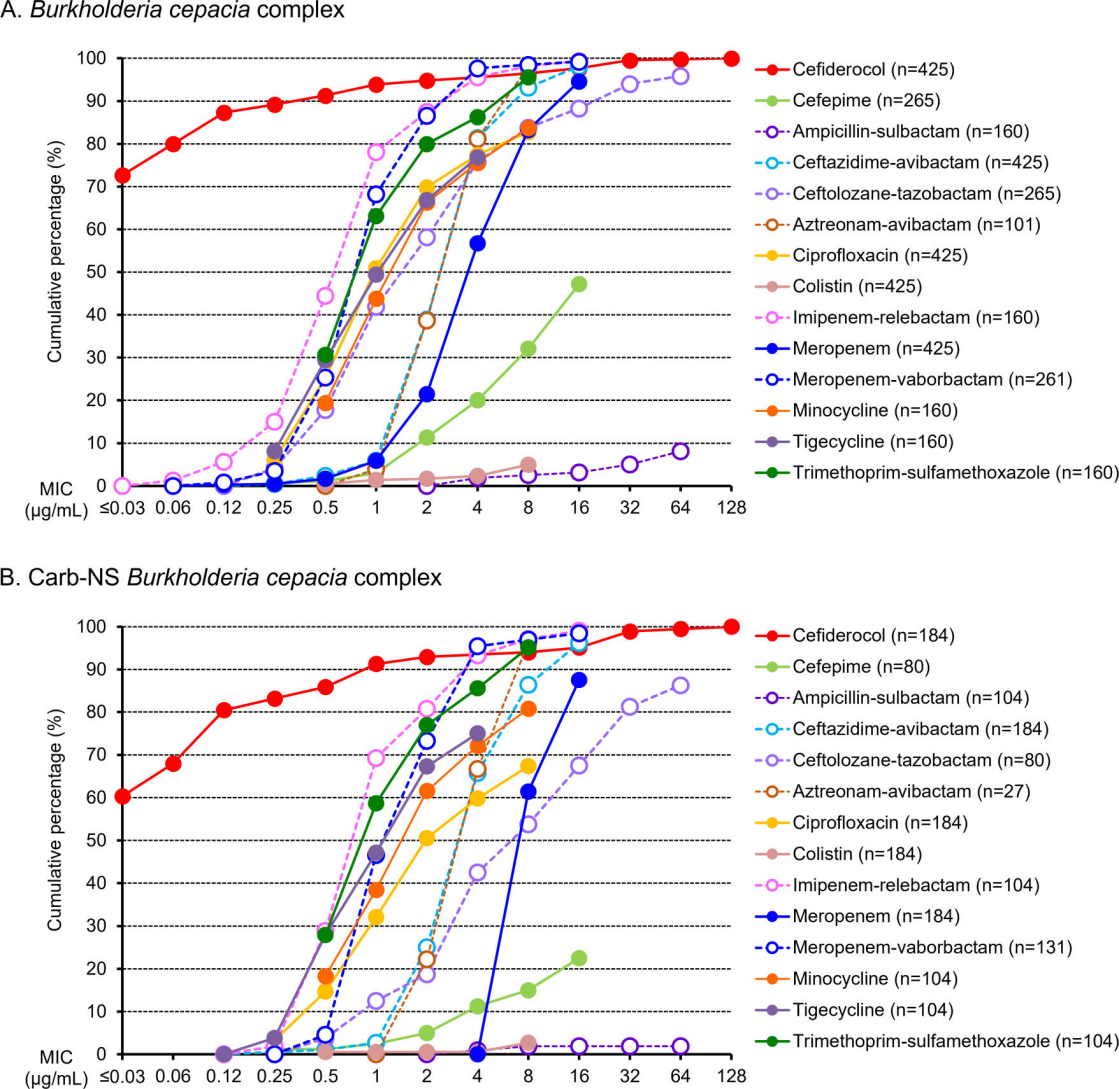
Cumulative percentage MIC distributions of cefiderocol and comparators against: (A) *Burkholderia cepacia* complex; (B) carbapenem-non-susceptible *Burkholderia cepacia* complex.

Against all 425 Bcc isolates, cefiderocol showed *in vitro* activity with MIC_50/90_ of ≤0.03/0.5 µg/mL, which were the lowest among the antibiotics tested (Table 3). Of 425 isolates, 22 (5.2%) were not susceptible to cefiderocol based on EUCAST PK/PD breakpoints (Table 3). According to cumulative MIC concentration distributions, cefiderocol was the most active of the agents tested against Bcc (Fig. 2A). Against 184 Carb-NS isolates, the MIC_50/90_ of cefiderocol was ≤0.03/1 µg/mL (Table 3), and 22 (12.0%) of these isolates had cefiderocol MIC >2 µg/mL. Cefiderocol inhibited the growth of 93.5% (172/184) of Carb-NS Bcc at a concentration of 4 µg/mL (Fig. 2B). Thirteen (7.1%) of 184 Carb-NS isolates were not susceptible to cefiderocol based on EUCAST PK/PD breakpoints (Table 3).

**TABLE 3:** *In vitro* activity of cefiderocol and comparators against *Burkholderia cepacia* complex collected in North America and Europe from 2014 to 2019 in the SIDERO-WT surveillance study [adapted from reference (14)]^*k*^

Phenotype (no. of isolates)	Antimicrobial agent	MIC (µg/mL)				Susceptibility rate (%)	
		*N*	Range	MIC_50_	MIC_90_	CLSI	EUCAST
All isolates	Cefiderocol	425	≤0.03 to 128	≤0.03	0.5	NA	94.8^*a*^
	Ampicillin-sulbactam	160	4 to >64	>64	>64	NA	0^*a*^
	Aztreonam-avibactam	101	1 to >8	4	8	NA	NA
	Cefepime	265	≤0.12 to >16	>16	>16	32.1^*b*^	20.0^*c*^
	Ceftazidime-avibactam	425	0.25 to >16	4	8	NA	93.2^*d*^
	Ceftolozane-tazobactam	265	0.25 to >64	2	32	NA	75.8^*c*^
	Ciprofloxacin	425	≤0.12 to >8	1	>8	50.8^*e*^	6.1^*f*^
	Colistin	425	≤0.25 to >8	>8	>8	NA	NA
	Imipenem-relebactam	160	0.06 to >16	1	4	NA	87.5^*a*^
	Meropenem	425	0.12 to >16	4	16	56.7^*g*^	21.4^*a*^
	Meropenem-vaborbactam	261	0.12 to >16	1	4	NA	98.5^*d*^
	Minocycline	160	≤0.25 to >8	2	>8	75.6^*g*^	NA
	Tigecycline	160	≤0.12 to >4	2	>4	NA	29.4^*h*^
	Trimethoprim-sulfamethoxazole	160	≤0.25 to >8	1	8	80.0^*i*^	NA
Meropenem non-susceptible^*j*^							
	Cefiderocol	184	≤0.03 to 128	≤0.03	1	NA	92.9^*a*^
	Ampicillin-sulbactam	104	4 to >64	>64	>64	NA	0^*a*^
	Aztreonam-avibactam	27	2 to >8	4	8	NA	NA
	Cefepime	80	≤0.06 to >16	>16	>16	15.0^*b*^	11.3^*c*^
	Ceftazidime-avibactam	184	0.25 to >16	4	16	NA	86.4^*d*^
	Ceftolozane-tazobactam	80	0.5 to >64	4	>64	NA	42.5^*c*^
	Ciprofloxacin	184	≤0.12 to >8	2	>8	32.1^*e*^	3.3^*f*^
	Colistin	184	≤0.25 to >8	>8	>8	NA	NA
	Imipenem-relebactam	104	0.25 to >16	1	4	NA	80.8^*a*^
	Meropenem	184	8 to >16	8	>16	0^*g*^	0^*a*^
	Meropenem-vaborbactam	131	0.5 to >16	2	4	NA	96.9^*d*^
	Minocycline	104	≤0.25 to >8	2	>8	72.1^*g*^	NA
	Tigecycline	104	0.25 to >4	2	>4	NA	27.9^*h*^
	Trimethoprim-sulfamethoxazole	104	≤0.25 to >8	1	8	76.9^*i*^	NA

^*a*^ EUCAST non-species PK/PD breakpoint: susceptible: ≤2 μg/mL.

^*b*^ CLSI breakpoint for other non-Enterobacterales: susceptible: ≤8 μg/mL.

^*c*^ EUCAST non-species PK/PD breakpoint: susceptible: ≤4 μg/mL.

^*d*^ EUCAST non-species PK/PD breakpoint: susceptible: ≤8 μg/mL.

^*e*^ CLSI breakpoint for other non-Enterobacterales: susceptible: ≤1 μg/mL.

^*f*^ EUCAST non-species PK/PD breakpoint: susceptible: ≤0.25 μg/mL.

^*g*^ CLSI breakpoint for *B. cepacia* complex: susceptible: ≤4 μg/mL.

^*h*^ EUCAST non-species PK/PD breakpoint: susceptible: ≤0.5 μg/mL.

^*i*^ CLSI breakpoint for *B. cepacia* complex: susceptible: ≤2 μg/mL.

^*j*^ Meropenem non-susceptible: meropenem MIC ≥8 μg/mL by CLSI.

^*k*^ CLSI, Clinical and Laboratory Standards Institute [18]; EUCAST, European Committee on Antimicrobial Susceptibility Testing [19]; NA, no breakpoint available.

### Molecular analysis of isolates with high cefiderocol MIC values

Whole-genome sequencing (WGS) on five *A. xylosoxidans* and one *Achromobacter* sp. isolates with high cefiderocol MIC values (MIC of 16 to >64 µg/mL) revealed that these six isolates had OXA-114-based or OXA-364 β-lactamases (Table S2). WGS on Bcc isolates with cefiderocol MICs of 16 to 128 µg/mL revealed that all 15 isolates had PenA β-lactamases (Table S3). Among these 15 Bcc isolates with high cefiderocol MIC values, 11 belonged to *Burkholderia multivorans*. By multilocus sequence type testing (MLST), no common sequence type (ST) was found in the five *A. xylosoxidans* and one *Achromobacter* sp. isolates or Bcc isolates with high cefiderocol MIC values.

The results of WGS and MLST characterization of the *A. xylosoxidans* and *B. cepacia* ATCC 25416 test isolates used for *in vivo* studies are shown in Tables S4 and S5, respectively. Both *A. xylosoxidans* isolates belonged to ST314 and had an OXA-114i β-lactamase gene detected, while AmpC, CTX-M-44, and OXA-18 β-lactamase genes were detected in the test *B. cepacia* ATCC 25416 isolate (Tables S4 and S5).

### *In vivo* activity in an immunocompetent rat lung infection model for *A. xylosoxidans* and *B. cepacia*

**Fig 3:**
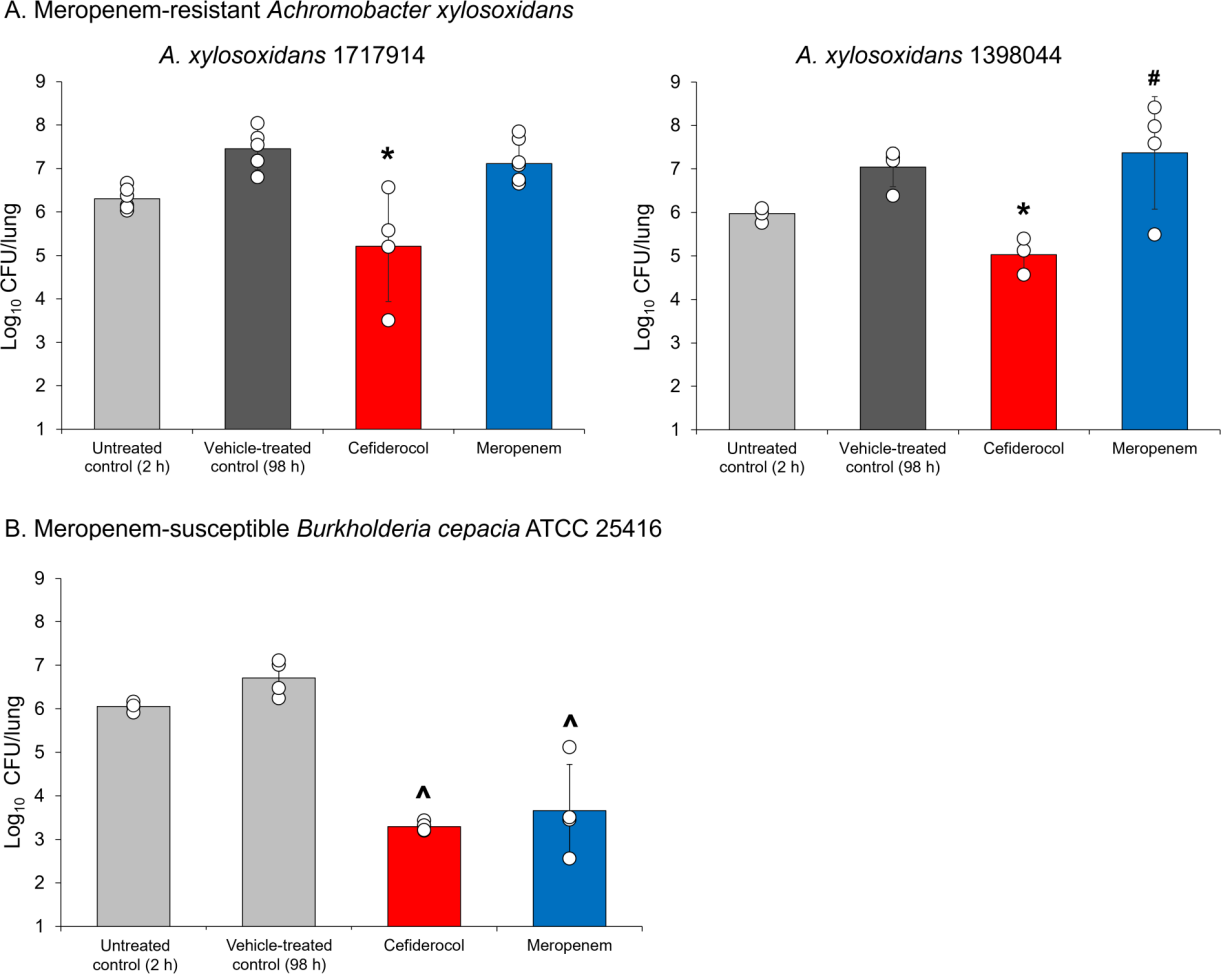
*In vivo* efficacy in an immunocompetent rat respiratory infection model of humanized cefiderocol against: (A) meropenem-resistant *Achromobacter xylosoxidans*; (B) meropenem-susceptible *Burkholderia cepacia* ATCC 25416. *Significant reduction (*P* < 0.05) vs vehicle-treated control, Welch’s *t* test. ^#^Significant difference (*P* < 0.05) vs cefiderocol, Welch’s *t* test. ^Significant reduction (*P* < 0.05) vs untreated control, Welch’s *t* test. Cefiderocol MIC: 0.5 µg/mL for *A. xylosoxidans* strain 1717914 and 2 µg/mL for *A. xylosoxidans* strain 1398044. Meropenem MIC: >16 µg/mL for *A. xylosoxidans* strain 1717914 and >16 µg/mL for *A. xylosoxidans* strain 1398044. Cefiderocol MIC: ≤0.03 µg/mL for *Burkholderia cepacia* ATCC 25416. Meropenem MIC: 4 µg/mL for *Burkholderia cepacia* ATCC 25416.

An immunocompetent rat lung infection model was used to assess the *in vivo* efficacy of cefiderocol against *A. xylosoxidans* and *B. cepacia* ATCC 25416. Antibiotics were administered at humanized doses to achieve plasma levels corresponding with the estimated plasma concentrations of the free drug in PK studies. Humanized cefiderocol dosing reduced the viable cell count by >2 log_10_ CFU/lung compared with vehicle-treated controls against both meropenem-resistant *A. xylosoxidans* 1717914 and *A. xylosoxidans* 1398044 (cefiderocol MIC: 0.5 and 2 µg/mL, respectively) (*P* < 0.05) (Fig. 3A). Meropenem showed no significant activity (meropenem MIC: >16 µg/mL for both strains).

Cefiderocol and meropenem showed bactericidal activity against a meropenem-susceptible *B. cepacia* ATCC 25416 strain (cefiderocol MIC: ≤0.03 µg/mL; meropenem MIC: 4 µg/mL), with 2.8 and 2.4 log_10_ CFU/lung decrease, respectively, compared with untreated control (*P* < 0.05) (Fig. 3B).

### *In vivo* activity in a neutropenic murine lung infection model for meropenem-resistant *A. xylosoxidans*

**Fig 4:**
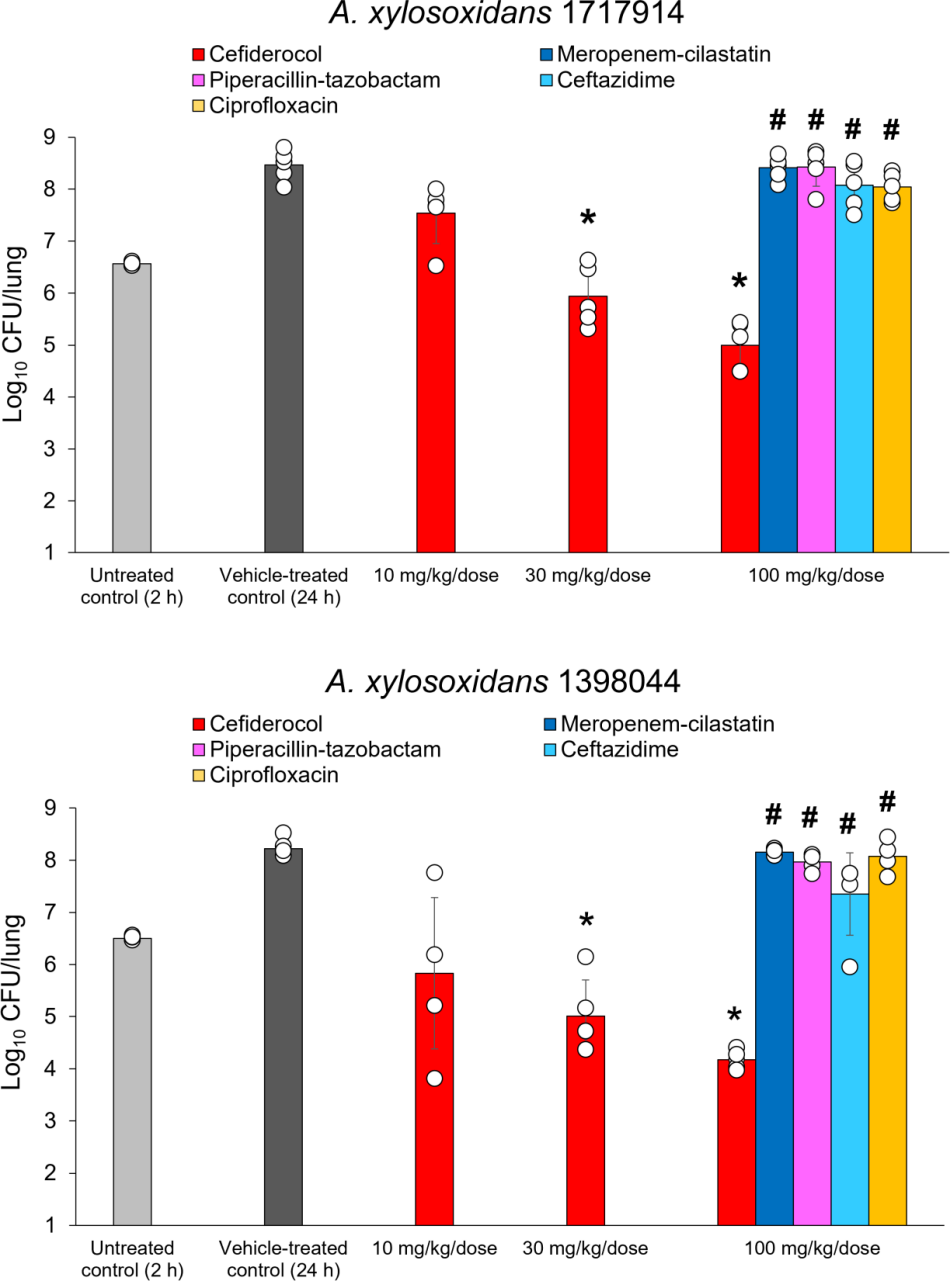
*In vivo* efficacy of cefiderocol against meropenem-resistant *Achromobacter xylosoxidans* in a neutropenic murine lung infection model. *Significant reduction (*P* < 0.05) vs untreated control, Dunnett’s multiple comparison test. ^#^Significant difference (*P* < 0.05) vs equivalent dose of cefiderocol, Welch’s *t* test. Cefiderocol MIC: 0.5 µg/mL for *A. xylosoxidans* strain 1717914 and 2 µg/mL for *A. xylosoxidans* strain 1398044. Meropenem MIC: >16 µg/mL for *A. xylosoxidans* strain 1717914 and >16 µg/mL for *A. xylosoxidans* strain 1398044.

Additional *in vivo* experiments were performed in a neutropenic murine lung infection model to assess the effectiveness of cefiderocol under neutropenic conditions against *A. xylosoxidans*. Cefiderocol caused >1.5 log_10_ decrease of viable bacteria in lungs at 100 mg/kg dose compared with untreated controls against both *A. xylosoxidans* 1717914 and *A. xylosoxidans* 1398044 (cefiderocol MICs: 0.5 and 2 µg/mL, respectively) (*P* < 0.05) (Fig. 4). No decrease of viable cells in lungs was observed for the comparators, which were used as control to confirm resistance, at 100 mg/kg (meropenem, piperacillin-tazobactam, ceftazidime, and ciprofloxacin MICs were >16, >64, >32, and >8 µg/mL, respectively) (Fig. 4).

## DISCUSSION

In this study, cefiderocol showed potent *in vitro* activity against *Achromobacter* spp. (MIC_90_ 0.5 µg/mL) and maintained its potency against isolates of carbapenem-non-susceptible *Achromobacter* spp. Only 11 *Achromobacter* spp. isolates overall, including six Carb-NS isolates, had cefiderocol MICs >2 µg/mL. In a neutropenic murine lung infection model and humanized PK immunocompetent rat lung infection model, cefiderocol showed significant bactericidal activity against two meropenem-resistant *A. xylosoxidans* strains compared with untreated controls (*P* < 0.05) and vehicle-treated controls (*P* < 0.05), respectively.

Cefiderocol also showed potent *in vitro* activity against Bcc (MIC_90_ 0.5 µg/mL) collected in the 5-year consecutive surveillance studies. Both cefiderocol and meropenem showed bactericidal activity in the immunocompetent rat lung infection model of meropenem-susceptible *B. cepacia* ATCC 25416, reflecting the *in vitro* activity observed in our study. This is reasonable, given that the %fT_>MIC_ achieved in this model exceeded the target required to cause 1-log_10_ reduction in murine thigh infection models (e.g., 88% for cefiderocol and 26% for meropenem against *A. baumannii*, respectively) (20). In addition, we have previously shown that, in immunocompetent rats infected with *S. maltophilia*, simulated humanized dosing of cefiderocol (2 g 3-h infusion) and meropenem (1 g 0.5-h infusion) resulted in plasma concentration-time profiles that were similar to human PK data for cefiderocol (21). By recreating the humanized PK exposure in this previous study, the %fT_>MIC_ was 100% for cefiderocol and 35% for meropenem, respectively (21).

WGS data on *Achromobacter* spp. and Bcc isolates with low cefiderocol susceptibility did not reveal the presence of known β-lactamases, specifically leading to cefiderocol resistance. It is interesting to note that the six ST *Achromobacter* spp. strains identified in our study were confirmed to be partly the same ST as previously reported strains from patients with CF that showed resistance to multiple antibacterial drugs (22). Three of the ST strains in our study showed resistance to the same drugs as the same ST strains obtained from patients with CF, while the remaining three ST strains in our study differed from the CF ST strains in the drugs to which they showed resistance. Nearly all Bcc isolates with high cefiderocol MIC values belonged to the same species (i.e., *B. multivorans*). Previous studies on cefiderocol susceptibility suggest that the presence of different resistance mechanisms, including mutations in iron transport-related proteins, defects in porin channels, and expression of certain β-lactamases, is needed to confer cefiderocol resistance in non-fermenter pathogens such as *A. baumannii* or *P. aeruginosa* (23–26). Thus, it may be possible that cefiderocol resistance in *Achromobacter* spp. and *Burkholderia* spp. or Bcc strains may emerge in the presence of similar mechanisms (23). However, data from our analyses are insufficient to confirm any role of mutations in iron transporters in cefiderocol-resistant isolates.

The activity of cefiderocol against *Achromobacter* spp. and *Burkholderia* spp. suggests that it may be a useful agent for the treatment of these infections, including pulmonary exacerbations in patients with CF infected with MDR organisms, or with rapid pulmonary decline, and without alternative treatment options (27). This is supported by data on cefiderocol use in individual patients or case series. In *A. xylosoxidans* infections, cefiderocol as monotherapy or combined with other antibiotics was effective in managing a series of heavily pretreated patients with CF (28) and a patient with severe MDR tracheobronchial infection and bacteremia (29), as well as clearing blood isolates from a non-Hodgkin lymphoma patient with aortic valve endocarditis (30). In a patient with CF and exacerbations due to pandrug-resistant and extensively drug-resistant *Achromobacter* spp., cefiderocol-based treatment restored lung function (31). Although there are few data on Bcc infections treated with cefiderocol, a recent case report showed that cefiderocol-based treatment improved symptoms and facilitated several months of clinical stability in a patient with CF, chronic Bcc colonization, and frequent exacerbations (32).

In view of the scarcity of published data on cefiderocol activity against *Achromobacter* spp. and *Burkholderia* spp. clinical isolates, the findings from the present study, the largest conducted comparing cefiderocol with other antibiotic agents in this setting and including a range of different species, provide valuable information on the efficacy of cefiderocol against these rare but difficult-to-treat non-fermenter pathogens. The limitations of the study include the lack of comparative *in vivo* data on the efficacy of cefiderocol and comparator antibiotics against meropenem-resistant Bcc corresponding to the *in vitro* susceptibility data. Bcc strains were not tested in the *in vivo* neutropenic murine infection model because cefiderocol MIC was very low (i.e., MIC ≤0.03 µg/mL); thus, the exposure level was expected to be too high to investigate any dose-response relationship. Additional *in vivo* experiments are still required with a range of *Achromobacter* spp. and *Burkholderia* spp. to better understand the potency of cefiderocol against these rare pathogens.

In conclusion, the results of our study suggest that cefiderocol could be a possible treatment option for RTIs caused by *Achromobacter* spp. and Bcc.

## MATERIALS AND METHODS

### Test organisms

*Achromobacter* spp. isolates were collected by International Health Management Associates, Inc. (IHMA, Schaumburg, IL, USA) from 39 countries (United States, Canada, France, Germany, Greece, Hungary, Italy, Latvia, Norway, Portugal, Russia, Serbia, Spain, United Kingdom, Argentina, Brazil, Colombia, Ecuador, Guatemala, Mexico, Panama, Puerto Rico, Venezuela, Egypt, South Africa, Hong Kong, Japan, Malaysia, South Korea, Taiwan, Thailand, Vietnam, Israel, Kuwait, Saudi Arabia, United Arab Emirates, Australia, New Zealand, and the Philippines) in 2015–2019. All isolates were obtained from specimens collected from patients with community-associated or hospital-associated infections from respiratory tract, blood, skin and skin structure, intra-abdominal, urinary tract, and unknown sources.

Bcc isolates were collected in North America (United States and Canada) and 11 European countries (United Kingdom, France, Germany, Italy, Spain, Czech Republic, Turkey, Russia, Hungary, Greece, and Sweden) in the 5 SIDERO-WT surveillance studies, sponsored by Shionogi & Co., Ltd. (Osaka, Japan), which were conducted annually from November 2014 to December 2019: SIDERO-WT-2014 (Year 1: 2014–2015), SIDERO-WT-2015 (Year 2: 2015–2016), SIDERO-WT-2016 (Year 3: 2016–2017), SIDERO-WT-2018 (Year 4: 2017–2018), and SIDERO-WT-2019 (Year 5: 2019). All isolates were shipped to IHMA for confirmation of identities using matrix-assisted laser desorption ionization-time of flight mass spectrometry (Bruker Daltonics, Billerica, MA, USA).

### Antimicrobial susceptibility testing

Clinical and Laboratory Standards Institute (CLSI)-defined broth microdilution susceptibility testing was performed at IHMA using custom in-house-prepared broth microdilution panels, and MICs were determined as described previously (14). Cefiderocol was tested in Chelex-treated iron-depleted cation-adjusted Mueller-Hinton broth (ID-CAMHB). All other antimicrobial agents were tested in standard CAMHB (BBL; Becton Dickinson, Sparks, MD, USA).

*Achromobacter* spp. MICs were determined with cefiderocol (Shionogi & Co., Ltd., Osaka, Japan), cefepime (USP), ceftazidime (USP), ceftazidime (USP) with avibactam (Biochempartner, Shanghai, China) at a fixed concentration of 4 µg/mL, ciprofloxacin (USP), colistin (USP), meropenem (USP), meropenem (USP) with vaborbactam (MedChemExpress, NJ, USA) at a fixed concentration of 8 µg/mL, imipenem (USP) with relebactam (MedChemExpress) at a fixed concentration of 4 µg/mL, tigecycline (Pfizer, NY, USA), trimethoprim (USP)-sulfamethoxazole (Sigma, MO, USA; 1:19).

MICs of piperacillin (Chem-Impex International, Inc., IL, USA) with tazobactam (Tokyo Chemical Industry Co., Ltd., Japan) at a fixed concentration of 4 µg/mL were also determined against *A. xylosoxidans* 1717914 and 1398044 strains used in the *in vivo* studies.

Bcc MICs were determined with cefiderocol (Shionogi & Co., Ltd.), ampicillin (USP)-sulbactam (USP), aztreonam (USP) with avibactam (Haoyuan ChemExpress Co., Ltd., Shanghai, China and Biochempartner) at a fixed concentration of 4 µg/mL, cefepime (USP), ceftazidime (USP) with avibactam (Haoyuan ChemExpress Co., Ltd., and Biochempartner) at a fixed concentration of 4 µg/mL, ceftolozane (Shionogi & Co., Ltd.)-tazobactam (USP) at a fixed concentration of 4 µg/mL, ciprofloxacin (USP), colistin (USP), meropenem (USP), meropenem (USP) with vaborbactam (MedChemExpress) at a fixed concentration of 8 µg/mL, imipenem (USP) with relebactam (MedChemExpress) at a fixed concentration of 4 µg/mL, minocycline (USP), tigecycline (Pfizer), trimethoprim (USP)-sulfamethoxazole (Sigma; 1:19).

### Susceptibility criteria

MIC interpretive criteria followed published guidelines established by the CLSI M100 Ed32 (18) and EUCAST Version 12.0 (19). Note: CLSI currently does not publish cefiderocol MIC breakpoints for *Achromobacter* spp. and Bcc.

For *Achromobacter* spp., susceptibility rates of cefepime (≤8 µg/mL), ceftazidime (≤8 µg/mL), ciprofloxacin (≤1 µg/mL), meropenem (≤4 µg/mL), and trimethoprim-sulfamethoxazole (≤2 µg/mL) were determined according to CLSI MIC interpretive criteria of other non-Enterobacterales. Additionally, susceptibility rates for meropenem (≤1 µg/mL) and trimethoprim-sulfamethoxazole (≤0.12 µg/mL) were determined according to EUCAST breakpoints for *A. xylosoxidans*. EUCAST non-species PK/PD breakpoints were used to determine susceptibility rates of cefiderocol (≤2 µg/mL), cefepime (≤4 µg/mL), ceftazidime (≤4 µg/mL), ceftazidime-avibactam (≤8 µg/mL), ciprofloxacin (≤0.25 µg/mL), imipenem-relebactam (≤2 µg/mL), meropenem-vaborbactam (≤8 µg/mL), and tigecycline (≤0.5 µg/mL).

For Bcc, susceptibility rates of meropenem (≤4 µg/mL), minocycline (≤4 µg/mL), and trimethoprim-sulfamethoxazole (≤2 µg/mL) were determined according to CLSI MIC interpretive criteria of Bcc. Susceptibility rates of cefepime (≤8 µg/mL) and ciprofloxacin (≤1 µg/mL) were determined according to CLSI MIC interpretive criteria of other non-Enterobacterales. Susceptibility rates of cefiderocol (≤2 µg/mL), cefepime (≤4 µg/mL), ceftazidime-avibactam (≤8 µg/mL), ceftolozane-tazobactam (≤4 µg/mL), ampicillin-sulbactam (≤2 µg/mL), ciprofloxacin (≤0.25 µg/mL), imipenem-relebactam (≤2 µg/mL), meropenem (≤2 µg/mL), meropenem-vaborbactam (≤8 µg/mL), and tigecycline (≤0.5 µg/mL) were determined according to EUCAST non-species PK/PD breakpoints.

In *in vitro* and *in vivo* studies, Carb-NS strains were defined as meropenem-non-susceptible strains (MIC ≥8 µg/mL) for both *A. xylosoxidans* and Bcc according to CLSI.

### *In vivo* efficacy

All studies with animals were approved by the Institutional Animal Care and Use Committee of Shionogi & Co., Ltd.

For the *in vivo* immunocompetent rat lung infection model for *A. xylosoxidans* and *B. cepacia* ATCC 25416, the model described by Nakamura et al. was used (21, 33). Specific-pathogen-free, 6-week-old, male Sprague Dawley rats (Charles River Laboratories, Yokohama, Japan) were used (*n* = 3–7/group for *A. xylosoxidans* and 4–5/group for *B. cepacia* ATCC 25416). Under anesthesia with a combined intramuscular (IM) injection of medetomidine, midazolam, and butorphanol, rats underwent surgery to insert a polyethylene tube into the jugular vein 5 days before bacterial inoculation. The rats were infected by intrabronchial inoculation of the bacterial suspension with 1% of molten nutrient agar. Inoculum sizes were *A. xylosoxidans* 1717914 and *A. xylosoxidans* 1398044: 2 × 10^6^ CFU/rat; and *B. cepacia* ATCC 25416: 5 × 10^6^ CFU/rat. Cefiderocol or meropenem (Sumitomo Pharma Co., Ltd., Osaka, Japan) was administered at 2 h post-infection via the implanted cannula into the jugular vein as controlled infusions. The free drug concentration in human plasma that was observed following intravenous administration of cefiderocol 2 g every 8 h (simulated 3-h infusion) and meropenem 1 g every 8 h (0.5-h infusion) was recreated in rats by computerized programs (SiMSP04, STLab Co., Ltd., Hitachinaka, Japan). Dosing continued for a total of 96 h. After exsanguination of rats under anesthesia with isoflurane inhalation, lungs were excised at 98 h post-infection, and the numbers of viable cells in lung tissue were counted. For the rat infection model, the lungs were removed from each rat and were homogenized with 5.4 mL of Mueller-Hinton broth. The viable bacterial cells were counted on Modified Drigalski agar plates after overnight incubation at 37°C. The lower limit of detection was 1.5 log_10_ CFU/g lung.

For the *in vivo* neutropenic mouse lung infection model for *A. xylosoxidans*, the model described by Nakamura et al. was used (20). Specific pathogen-free, 5-week-old, male Jcl:ICR mice (weight 17–20 g) were obtained from CLEA Japan, Inc. (Tokyo, Japan). Mice were rendered neutropenic by intraperitoneal injection of cyclophosphamide (Shionogi & Co., Ltd., Osaka, Japan) 150 and 100 mg/kg at 4 and 1 day before infection, respectively. Mice were anesthetized by IM injection of a mixture of medetomidine, midazolam, and butorphanol, and then infected by intranasal instillation of 0.07 mL of bacterial suspension with saline. The challenge dose was 2–3 × 10^6^ CFU/mouse. Antibiotics (i.e., cefiderocol, meropenem-cilastatin, piperacillin-tazobactam, ceftazidime, and ciprofloxacin) were administered subcutaneously at 2, 5, and 8  h post-infection (*n* = 5/group). Meropenem was administered with cilastatin (MedChemExpress) at the same dosage (i.e., 100 mg/kg) to minimize meropenem degradation by murine dihydropeptidase-1 (34). Control mice were treated with 0.9% saline. At 24 h post-infection, lungs were excised, and the numbers of viable cells in lung tissue were counted. For the mouse infection model, the lungs were removed from each mouse and were homogenized with 1.8 mL of Mueller-Hinton broth. The viable bacterial cells were counted on Modified Drigalski agar plates after overnight incubation at 37°C. The lower limit of detection was 1.0 log_10_ CFU/g lung.

### WGS analysis

WGS analysis was performed against *Achromobacter* spp. and Bcc strains with low cefiderocol susceptibility (cefiderocol MIC >8 µg/mL) and against the clinical isolates used in the *in vivo* studies.

*Achromobacter* spp.: WGS analysis was conducted at Shionogi & Co., Ltd. Genomic DNA from each bacterial sample was extracted using the Illustra bacteria genomic Prep Mini Spin Kit (GE Healthcare UK Limited, Chalfont St Giles, UK). As described previously, WGS was performed on an Illumina MiSeq system (35), and all analyses were carried out using the CLC Genomics Workbench version 11.0.1 (Qiagen) and Pipeline Pilot version 9.5.0.831 (Dassault Systèmes Biovia, San Diego, CA, USA) to identify the β-lactamase genes (36). For MLST determination, the sequences of seven housekeeping genes (*nusA, rpoB, eno, gltB, lepA, nuoL,* and *nrdA*) were investigated using the PubMLST typing database.

*B. cepacia* complex: WGS analysis was conducted at IHMA (Schaumburg, IL, USA). Genomic DNA from each bacterial sample was extracted using the DNeasy Ultraclean Microbial Extraction Kit (Qiagen, MD, USA). As described previously, WGS was performed on an Illumina Hiseq system, and all analyses were carried out using the CLC Genomics Workbench v. 20 (Qiagen) to identify β-lactamase genes (37). For MLST determination, the sequences of seven housekeeping genes (*atpD, gltB, gyrB, recA, lepA, phaC,* and *trpB*) were investigated using the PubMLST typing database.

### Statistical analysis

Dunnett’s multiple comparison test was used to test differences between multiple doses of cefiderocol treatment and untreated baseline control groups in the number of viable cells. Welch’s *t* test was used to compare differences between two groups in the number of viable cells. In both cases, the two-sided *P* value of <0.05 was considered to be statistically significant.

## Data Availability

All analyzed data collected in the study are included in this manuscript. Data obtained in whole-genome sequencing are deposited in the Japanese DDBJ database and can be accessed at https://www.ddbj.nig.ac.jp/biosample/index-e.html. List of accession numbers and crosslinks to the NIH NCBI database can be found in Table S6.

## SUPPLEMENTAL MATERIAL

Supplemental file 1 aac.00346-23-s0001.docx Tables S1 to S5.

Supplemental file 2 aac.00346-23-s0002.xlsx Table S6.

ASM does not own the copyrights to Supplemental Material that may be linked to, or accessed through, an article. The authors have granted ASM a non-exclusive, world-wide license to publish the Supplemental Material files. Please contact the corresponding author directly for reuse.
