# Supplementary material for: In vitro and in vivo activity of cefiderocol against Achromobacter spp. and Burkholderia cepacia complex, including carbapenem-non-susceptible isolates
Source: Antimicrob Agents Chemother. 2023 Nov 16;67(12):e00346-23. doi: 10.1128/aac.00346-23 (PMC10720420; doi:10.1128/aac.00346-23)
Supplement: Supplemental file 1 — Tables S1 to S5. [file aac.00346-23-s0001.docx]

**SUPPLEMENTARY TABLE 1** Geographical origin of overall, carbapenem-susceptible and carbapenem non-susceptible *Achromobacter* spp. and *Burkholderia cepacia* complex infections

**SUPPLEMENTARY TABLE 2** WGS and MLST characterization of *Achromobacter xylosoxidans* and *Achromobacter* spp. isolates with low cefiderocol susceptibility

**SUPPLEMENTARY TABLE 3** WGS and MLST characterization of *Burkholderia cepacia* complex isolates with low cefiderocol susceptibility

**SUPPLEMENTARY TABLE 4** WGS and MLST characterization of *Achromobacter* *xylosoxidans* strains used for *in vivo* studies

**SUPPLEMENTARY TABLE 5** WGS and MLST characterization of *Burkholderia cepacia* strain used for *in vivo* studies

**SUPPLEMENTARY TABLE 6** Accession numbers for *Burkholderia multivorans*, *B. vietnamiensis*, *B. cenocepacia*, *B. cepacia*, *Achromobacter xylosoxidans*, *Achromobacter* sp. isolates

**SUPPLEMENTARY TABLE 1** Geographical origin of overall, carbapenem-susceptible and carbapenem non-susceptible *Achromobacter* spp. and *Burkholderia* *cepacia* complex infections

| **Region** | **Total (%)** | **Carbapenem-susceptible (%)*^a^*** | **Carbapenem  non-susceptible (%)*^b^*** |
| --- | --- | --- | --- |
| ***Achromobacter* spp.** | | | |
| **All** | **334 (100)** | **282 (84.4^c^)** | **52 (15.6^c^)** |
| North America | 177 (53.0) | 143 (50.7) | 34 (65.4) |
| Latin America | 46 (13.8) | 40 (14.2) | 6 (11.5) |
| Asia | 45 (13.5) | 44 (15.6) | 1 (1.9) |
| Europe | 34 (10.2) | 30 (10.6) | 4 (7.7) |
| South Pacific | 24 (7.2) | 18 (6.4) | 6 (11.5) |
| Middle East | 6 (1.8) | 5 (1.8) | 1 (1.9) |
| Africa | 2 (0.6) | 2 (0.7) | 0 (0) |
| ***Burkholderia* *cepacia* complex** | | | |
| **All** | **425 (100)** | **241 (56.7^c^)** | **184 (43.3^c^)** |
| United States | 155 (36.5) | 90 (37.3) | 65 (35.3) |
| Spain | 51 (12.0) | 20 (8.3) | 31 (16.8) |
| Canada | 41 (9.6) | 20 (8.3) | 21 (11.4) |
| Czech Republic | 39 (9.2) | 24 (10.0) | 15 (8.2) |
| Turkey | 31 (7.3) | 19 (7.9) | 12 (6.5) |
| Italy | 24 (5.6) | 16 (6.6) | 8 (4.3) |
| France | 18 (4.2) | 9 (3.7) | 9 (4.9) |
| Russia | 18 (4.2) | 17 (7.1) | 1 (0.5) |
| United Kingdom | 18 (4.2) | 6 (2.5) | 12 (6.5) |
| Germany | 15 (3.5) | 8 (3.3) | 7 (3.8) |
| Hungary | 11 (2.6) | 8 (3.3) | 3 (1.6) |
| Greece | 3 (0.7) | 3 (1.2) | 0 (0) |
| Sweden | 1 (0.2) | 1 (0.4) | 0 (0) |

*^a^*As a proportion of all carbapenem-susceptible isolates, unless otherwise specified.

*^b^*As a proportion of all carbapenem non-susceptible isolates, unless otherwise specified.

*^c^*As a proportion of the total number of isolates.

**SUPPLEMENTARY TABLE 2** WGS and MLST characterization of *Achromobacter xylosoxidans* and *Achromobacter* spp. isolates with low cefiderocol susceptibility

| **Organism** | **Isolate**  **(country)** | **MIC (µg/mL)** | | | | | | | | | | | **β-lactamase** | **MLST** |
| --- | --- | --- | --- | --- | --- | --- | --- | --- | --- | --- | --- | --- | --- | --- |
|  |  | **FDC** | **FEP** | **CAZ** | **CZA** | **CIP** | **CST** | **I-R** | **MEM** | **MVB** | **TGC** | **SXT** |  |  |
| *A. xylosoxidans* | 1221102  (UK) | 16 | >32 | 16 | 8 | 8 | >8 | 2 | 2 | 2 | 1 | ≤0.25 | OXA-114c | 175 |
| *A. xylosoxidans* | 1219416  (USA) | 16 | >32 | >32 | 8 | >8 | >8 | >16 | >16 | >16 | 8 | 4 | OXA-114i-like | 28 |
| *A. xylosoxidans* | 1377193  (Canada) | 32 | >32 | 8 | 8 | >8 | 4 | 1 | 2 | 2 | 4 | 1 | OXA-364 | ND |
| *Achromobacter sp.* | 1998979  (USA) | 32 | >32 | >32 | >16 | >8 | >8 | >16 | >16 | >16 | 1 | 16 | OXA-114i | ND |
| *A. xylosoxidans* | 1373226  (Canada) | >64 | >32 | >32 | >16 | >8 | >8 | 2 | 8 | 8 | 4 | 1 | OXA-114 | 176 |
| *A. xylosoxidans* | 1377194  (Canada) | >64 | >32 | 32 | 16 | >8 | 4 | 1 | 2 | 2 | 2 | 8 | OXA-364 | ND |

Abbreviations: CAZ, ceftazidime; CIP, ciprofloxacin; CST, colistin; CZA, ceftazidime-avibactam; FDC, cefiderocol; FEP, cefepime; I-R, imipenem-relebactam; MEM, meropenem; MLST, multilocus sequence typing; MVB, meropenem-vaborbactam; ND, undefined MLST because the corresponding gene sequence was not registered in PubMLST; SXT, trimethoprim-sulfamethoxazole; TGC, tigecycline; WGS, whole-genome sequencing.

**SUPPLEMENTARY TABLE 3** WGS and MLST characterization of *Burkholderia cepacia* complex isolates with low cefiderocol susceptibility

| **Organism** | **Isolate**  **(country)** | **MIC (µg/mL)** | | | | | | | | | | | | | | **β-lactamase** | **MLST** |
| --- | --- | --- | --- | --- | --- | --- | --- | --- | --- | --- | --- | --- | --- | --- | --- | --- | --- |
|  |  | **FDC** | **SAM** | **ATM-AVI** | **FEP** | **CZA** | **C/T** | **CIP** | **CST** | **I-R** | **MEM** | **MVB** | **MIN** | **TGC** | **SXT** |  |  |
| *B. cepacia* | 1219352  (USA) | 16 | NA | NA | 16 | 4 | 4 | 8 | >8 | NA | 2 | NA | NA | NA | NA | PenA-type | ND |
| *B. cenocepacia* | 1915491  (Canada) | 16 | NA | >8 | >16 | 8 | 16 | >8 | >8 | NA | 16 | 4 | NA | NA | NA | Unnamed Class D; PenA-type | 1076 |
| *B. cenocepacia* | 2085407  (Italy) | 16 | >64 | NA | >16 | 4 | NA | >8 | >8 | 2 | 4 | 1 | >8 | >4 | >8 | PenA-type | 46 |
| *B. multivorans* | 2135512  (France) | 16 | >64 | NA | 16 | 4 | NA | 4 | >8 | 1 | 4 | 2 | 2 | 4 | 1 | PenA-type | 287 |
| *B. vietnamensis* | 2085409 (Italy) | 16 | >64 | NA | >16 | 8 | NA | >8 | >8 | 8 | 8 | 2 | 8 | >4 | 8 | PenA-type | ND |
| *B. multivorans* | 1484350  (USA) | 32 | NA | NA | >16 | 4 | 4 | >8 | >8 | NA | 8 | NA | NA | NA | NA | PenA-type | 117 |
| *B. multivorans* | 1543270  (UK) | 32 | NA | NA | >16 | 4 | 32 | >8 | >8 | NA | >16 | NA | NA | NA | NA | PenA-like partial CDS | 374 |
| *B. multivorans* | 1543273  (UK) | 32 | NA | NA | >16 | 8 | 32 | >8 | >8 | NA | 16 | NA | NA | NA | NA | PenA-like partial CDS | 1088 |
| *B. multivorans* | 1910640  (Spain) | 32 | NA | 8 | 4 | 16 | 64 | >8 | >8 | NA | >16 | 16 | NA | NA | NA | PenA-type | 814 |
| *B. multivorans* | 1908122  (USA) | 32 | NA | 8 | 8 | 4 | 1 | 8 | >8 | NA | 1 | 1 | NA | NA | NA | PenA-type | ND |
| *B. multivorans* | 2088439  (USA) | 32 | >64 | NA | >16 | 2 | NA | >8 | >8 | 8 | 16 | 4 | 8 | >4 | 4 | Un-named Class D; PenA-type | 622 |
| *B. multivorans* | 2081214  (Spain) | 32 | >64 | NA | >16 | 4 | NA | >8 | >8 | 16 | >16 | 4 | 8 | >4 | >8 | PenA-type | 814 |
| *B. multivorans* | 2100641  (Germany) | 32 | 8 | NA | >16 | 4 | NA | >8 | >8 | 8 | >16 | >16 | 8 | >4 | 1 | PenA-type | 1023 |
| *B. multivorans* | 1557705  (USA) | 64 | NA | NA | >16 | 16 | 8 | >8 | >8 | NA | 16 | NA | NA | NA | NA | PenA-type | 17 |
| *B. multivorans* | 1919566  (USA) | 128 | NA | 8 | >16 | 8 | >64 | >8 | >8 | NA | 16 | 4 | NA | NA | NA | PenA-type | 18 |

Abbreviations: ATM-AVI, aztreonam-avibactam; CIP, ciprofloxacin; CST, colistin; C/T, ceftolozane/tazobactam; CZA, ceftazidime-avibactam; FDC, cefiderocol; FEP, cefepime; IHMA, International Health Management Associates; I-R, imipenem-relebactam; MEM, meropenem; MIN, minocycline; MLST, multilocus sequence typing; MVB, meropenem-vaborbactam; NA, not available; ND, undefined MLST because the corresponding gene sequence was not registered in PubMLST; SAM, ampicillin-sulbactam; SXT, trimethoprim-sulfamethoxazole; TGC, tigecycline; WGS, whole-genome sequencing.

**SUPPLEMENTARY TABLE 4** WGS and MLST characterization of *Achromobacter* *xylosoxidans* strains used for *in vivo* studies

| **Test isolate** | **MIC (µg/mL)** | | | | | | | | | | | **β-lactamase** | **MLST** |
| --- | --- | --- | --- | --- | --- | --- | --- | --- | --- | --- | --- | --- | --- |
|  | **FDC** | **FEP** | **CAZ** | **CZA** | **CIP** | **CST** | **I-R** | **MEM** | **MVB** | **TGC** | **SXT** |  |  |
| *A. xylosoxidans*  1717914 | 0.5 | >32 | >32 | 16 | >8 | 1 | >16 | >16 | >16 | 4 | 8 | OXA-114i | 314 |
| *A. xylosoxidans*  1398044 | 2 | >32 | >32 | 16 | >8 | 0.5 | >16 | >16 | >16 | 4 | 8 | OXA-114i | 314 |

Abbreviations: CAZ, ceftazidime; CIP, ciprofloxacin; CST, colistin; CZA, ceftazidime-avibactam; FDC, cefiderocol; FEP, cefepime; I-R, imipenem-relebactam; MEM, meropenem; MLST, multilocus sequence typing; MVB, meropenem-vaborbactam; SXT, trimethoprim-sulfamethoxazole; TGC, tigecycline; WGS, whole-genome sequencing.

**SUPPLEMENTARY TABLE 5** WGS and MLST characterization of *Burkholderia cepacia* strain used for *in vivo* studies

| **Test isolate** | **MIC (µg/mL)** | | **β-lactamase** | **MLST** |
| --- | --- | --- | --- | --- |
|  | **FDC** | **MEM** |  |  |
| *B. cepacia*  ATCC 25416 | ≤0.03 | 4 | AmpC;  CTX-M-44 (Toho-1);  OXA-18 | 10 |

Abbreviations: FDC, cefiderocol; MEM, meropenem; MLST, multilocus sequence typing; WGS, whole-genome sequencing.

β-lactamase information source: ATCC Genome Portal (https://genomes.atcc.org/genomes/fdb6ee7d7a1c4ffc?tab=annotations-tab)
MLST information source: <https://www.burkholderia.com/strain/show/2873>
